# THE CONCISE GUIDE TO PHARMACOLOGY 2017/18: Overview

**DOI:** 10.1111/bph.13882

**Published:** 2017-10-21

**Authors:** Stephen PH Alexander, Eamonn Kelly, Neil V Marrion, John A Peters, Elena Faccenda, Simon D Harding, Adam J Pawson, Joanna L Sharman, Christopher Southan, O Peter Buneman, John A Cidlowski, Arthur Christopoulos, Anthony P Davenport, Doriano Fabbro, Michael Spedding, Jörg Striessnig, Jamie A Davies, CGTP Collaborators, M‐P Abbracchio, R Aldrich, K Al‐Hosaini, TV Arumugam, B Attali, M Bäck, NM Barnes, R Bathgate, PM Beart, E Becirovic, B Bettler, M Biel, NJ Birdsall, V Blaho, D Boison, H Bräuner‐osborne, S Bröer, C Bryant, G Burnstock, G Calo, WA Catterall, S Ceruti, SL Chan, KG Chandy, P Chazot, N Chiang, JJ Chun, J‐J Chung, DE Clapham, L Clapp, MA Connor, HM Cox, P Davies, PA Dawson, P Decaen, G Dent, P Doherty, SD Douglas, ML Dubocovich, TM Fong, CJ Fowler, A Frantz, P Fuller, M Fumagalli, AH Futerman, RR Gainetdinov, MA Gershengorn, A Goldin, SAN Goldstein, C Goudet, K Gregory, S Grissmer, AL Gundlach, B Hagenbuch, J Hamann, JR Hammond, JC Hancox, J Hanson, I Hanukoglu, DL Hay, AJ Hobbs, AN Hollenberg, ND Holliday, D Hoyer, AP Ijzerman, KI Inui, AJ Irving, S Ishii, KA Jacobson, LY Jan, MF Jarvis, R Jensen, R Jockers, LK Kaczmarek, Y Kanai, S Karnik, S Kellenberger, S Kemp, C Kennedy, ID Kerr, Y Kihara, J Kukkonen, D Larhammar, K Leach, D Lecca, S Leeman, J Leprince, SJ Lolait, D Macewan, JJ Maguire, F Marshall, J Mazella, CA Mcardle, MC Michel, LJ Miller, V Mitolo, H Mizuno, PN Monk, B Mouillac, PM Murphy, J‐L Nahon, J Nerbonne, CG Nichols, X Norel, S Offermanns, LG Palmer, MA Panaro, A Papapetropoulos, E Perez‐reyes, RG Pertwee, S Pintor, JR Pisegna, LD Plant, DR Poyner, ER Prossnitz, S Pyne, R Ramachandran, D Ren, P Rondard, C Ruzza, H Sackin, G Sanger, MC Sanguinetti, L Schild, H Schiöth, G Schulte, S Schulz, DL Segaloff, CN Serhan, KD Singh, PA Slesinger, TP Snutch, CG Sobey, G Stewart, LA Stoddart, RJ Summers, C Szabo, D Thwaites, L Toll, JS Trimmer, S Tucker, H Vaudry, T Verri, J‐P Vilargada, SA Waldman, DT Ward, SG Waxman, AD Wei, GB Willars, SS Wong, TM Woodruff, H Wulff, RD Ye, Y Yung, J‐M Zajac

**Affiliations:** ^1^ School of Life Sciences University of Nottingham Medical School Nottingham NG7 2UH UK; ^2^ School of Physiology, Pharmacology and Neuroscience University of Bristol Bristol BS8 1TD UK; ^3^ Neuroscience Division, Medical Education Institute Ninewells Hospital and Medical School, University of Dundee Dundee DD1 9SY UK; ^4^ Centre for Integrative Physiology University of Edinburgh Edinburgh EH8 9XD UK; ^5^ Laboratory for Foundations of Computer Science, School of Informatics University of Edinburgh Edinburgh EH8 9LE United Kingdom; ^6^ National Institute of Environmental Health Sciences, National Institutes of Health Department of Health and Human Services Research Triangle Park NC 27709 USA; ^7^ Monash Institute of Pharmaceutical Sciences and Department of Pharmacology Monash University Parkville Victoria 3052 Australia; ^8^ Clinical Pharmacology Unit University of Cambridge Cambridge CB2 0QQ UK; ^9^ PIQUR Therapeutics Basel 4057 Switzerland; ^10^ Spedding Research Solutions SARL Le Vésinet 78110 France; ^11^ Pharmacology and Toxicology Institute of Pharmacy University of Innsbruck A‐6020 Innsbruck Austria

## Abstract

The Concise Guide to PHARMACOLOGY 2017/18 is the third in this series of biennial publications. This version provides concise overviews of the key properties of nearly 1800 human drug targets with an emphasis on selective pharmacology (where available), plus links to an open access knowledgebase of drug targets and their ligands (www.guidetopharmacology.org), which provides more detailed views of target and ligand properties. Although the Concise Guide represents approximately 400 pages, the material presented is substantially reduced compared to information and links presented on the website. It provides a permanent, citable, point‐in‐time record that will survive database updates. The full contents of this section can be found at http://onlinelibrary.wiley.com/doi/10.1111/bph.13882/full. In addition to this overview, in which are identified ‘Other protein targets’ which fall outside of the subsequent categorisation, there are eight areas of focus: G protein‐coupled receptors, ligand‐gated ion channels, voltage‐gated ion channels, other ion channels, nuclear hormone receptors, catalytic receptors, enzymes and transporters. These are presented with nomenclature guidance and summary information on the best available pharmacological tools, alongside key references and suggestions for further reading. The landscape format of the Concise Guide is designed to facilitate comparison of related targets from material contemporary to mid‐2017, and supersedes data presented in the 2015/16 and 2013/14 Concise Guides and previous Guides to Receptors and Channels. It is produced in close conjunction with the Nomenclature Committee of the Union of Basic and Clinical Pharmacology (NC‐IUPHAR), therefore, providing official IUPHAR classification and nomenclature for human drug targets, where appropriate.

## Table of contents

## S1 Overview

S6 Other Protein Targets

S6 Adiponectin receptors

S7 Blood coagulation components

S8 Non‐enzymatic BRD containing proteins

S8 Carrier proteins

S9 CD molecules

S10 Methyllysine reader proteins

S11 Fatty acid‐binding proteins

S13 Notch receptors

S13 Regulators of G protein Signaling (RGS) proteins

S14 Sigma receptors

S15 Tubulins

**S17 G protein‐coupled receptors**

S19 Orphan and other 7TM receptors

S19 Class A Orphans

S28 Class C Orphans

S28 Taste 1 receptors

S29 Taste 2 receptors

S30 TM proteins

S31 5‐Hydroxytryptamine receptors

S34 Acetylcholine receptors (muscarinic)

S36 Adenosine receptors

S37 Adhesion Class GPCRs

S39 Adrenoceptors

S43 Angiotensin receptors

S44 Apelin receptor

S45 Bile acid receptor

S46 Bombesin receptors

S47 Bradykinin receptors

S48 Calcitonin receptors

S50 Calcium‐sensing receptor

S51 Cannabinoid receptors

S52 Chemerin receptor

S53 Chemokine receptors

S57 Cholecystokinin receptors

S58 Class Frizzled GPCRs

S59 Complement peptide receptors

S60 Corticotropin‐releasing factor receptors

S61 Dopamine receptors

S63 Endothelin receptors

S64 G protein‐coupled estrogen receptor

S65 Formylpeptide receptors

S66 Free fatty acid receptors

S67 GABA_*B*_ receptors

S69 Galanin receptors

S70 Ghrelin receptor

S71 Glucagon receptor family

S72 Glycoprotein hormone receptors

S73 Gonadotrophin‐releasing hormone receptors

S75 GPR18, GPR55 and GPR119

S76 Histamine receptors

S77 Hydroxycarboxylic acid receptors

S78 Kisspeptin receptor

S79 Leukotriene receptors

S81 Lysophospholipid (LPA) receptors

S82 Lysophospholipid (S1P) receptors

S83 Melanin‐concentrating hormone receptors

S84 Melanocortin receptors

S85 Melatonin receptors

S86 Metabotropic glutamate receptors

S88 Motilin receptor

S89 Neuromedin U receptors

S90 Neuropeptide FF/neuropeptide AF receptors

S91 Neuropeptide S receptor

S92 Neuropeptide W/neuropeptide B receptors

S93 Neuropeptide Y receptors

S94 Neurotensin receptors

S95 Opioid receptors

S97 Orexin receptors

S98 Oxoglutarate receptor

S98 P2Y receptors

S101 Parathyroid hormone receptors

S101 Platelet‐activating factor receptor

S102 Prokineticin receptors

S103 Prolactin‐releasing peptide receptor

S104 Prostanoid receptors

S106 Proteinase‐activated receptors

S107 QRFP receptor

S108 Relaxin family peptide receptors

S110 Somatostatin receptors

S111 Succinate receptor

S111 Tachykinin receptors

S113 Thyrotropin‐releasing hormone receptors

S113 Trace amine receptor

S114 Urotensin receptor

S115 Vasopressin and oxytocin receptors

S117 VIP and PACAP receptors

**S130 Ligand‐gated ion channels**

S131 5‐HT_3_ receptors

S133 Acid‐sensing (proton‐gated) ion channels (ASICs)

S135 Epithelial sodium channels (ENaC)

S137 GABA_*A*_ receptors

S142 Glycine receptors

S145 Ionotropic glutamate receptors

S150 IP_3_ receptor

S151 Nicotinic acetylcholine receptors

S154 P2X receptors

S156 ZAC

**S160 Voltage‐gated ion channels**

S161 CatSper and Two‐Pore channels

S163 Cyclic nucleotide‐regulated channels

S164 Potassium channels

S165 Calcium‐ and sodium‐activated potassium channels

S166 Inwardly rectifying potassium channels

S169 Two P domain potassium channels

S171 Voltage‐gated potassium channels

S175 Ryanodine receptor

S176 Transient Receptor Potential channels

S186 Voltage‐gated calcium channels

S189 Voltage‐gated proton channel

S190 Voltage‐gated sodium channels

**S195 Other ion channels**

S196 Aquaporins

S197 Chloride channels

S197 ClC family

S199 CFTR

S200 Calcium activated chloride channel

S201 Maxi chloride channel

S202 Volume regulated chloride channels

S204 Connexins and Pannexins

S206 Sodium leak channel, non‐selective

**S208 Nuclear hormone receptors**

S209 1A. Thyroid hormone receptors

S210 1B. Retinoic acid receptors

S210 1C. Peroxisome proliferator‐activated receptors

S211 1D. Rev‐Erb receptors

S212 1F. Retinoic acid‐related orphans

S213 1H. Liver X receptor‐like receptors

S214 1I. Vitamin D receptor‐like receptors

S214 2A. Hepatocyte nuclear factor‐4 receptors

S215 2B. Retinoid X receptors

S216 2C. Testicular receptors

S216 2E. Tailless‐like receptors

S217 2F. COUP‐TF‐like receptors

S218 3B. Estrogen‐related receptors

S218 4A. Nerve growth factor IB‐like receptors

S219 5A. Fushi tarazu F1‐like receptors

S220 6A. Germ cell nuclear factor receptors

S220 0B. DAX‐like receptors

S221 Steroid hormone receptors

S221 3A. Estrogen receptors

S222 3C. 3‐Ketosteroid receptors

**S225 Catalytic receptors**

S226 Cytokine receptor family

S227 IL‐2 receptor family

S229 IL‐3 receptor family

S230 IL‐6 receptor family

S231 IL‐12 receptor family

S232 Prolactin receptor family

S233 Interferon receptor family

S234 IL‐10 receptor family

S235 Immunoglobulin‐like family of IL‐1 receptors

S236 IL‐17 receptor family

S237 GDNF receptor family

S237 Integrins

S241 Natriuretic peptide receptor family

S242 Pattern recognition receptors

S243 Toll‐like receptor family

S244 NOD‐like receptor family

S246 Receptor tyrosine kinases (RTKs)

S247 Type I RTKs: ErbB (epidermal growth factor) receptor family

S248 Type II RTKs: Insulin receptor family

S249 Type III RTKs: PDGFR, CSFR, Kit, FLT3 receptor family

S250 Type IV RTKs: VEGF (vascular endothelial growth factor) receptor family

S251 Type V RTKs: FGF (fibroblast growth factor) receptor family

S252 Type VI RTKs: PTK7/CCK4

S252 Type VII RTKs: Neurotrophin receptor/Trk family

S253 Type VIII RTKs: ROR family

S254 Type IX RTKs: MuSK

S254 Type X RTKs: HGF (hepatocyte growth factor) receptor family

S255 Type XI RTKs: TAM (TYRO3‐, AXL‐ and MER‐TK) receptor family

S255 Type XII RTKs: TIE family of angiopoietin receptors

S256 Type XIII RTKs: Ephrin receptor family

S257 Type XIV RTKs: RET

S257 Type XV RTKs: RYK

S258 Type XVI RTKs: DDR (collagen receptor) family

S258 Type XVII RTKs: ROS receptors

S259 Type XVIII RTKs: LMR family

S259 Type XIX RTKs: Leukocyte tyrosine kinase (LTK) receptor family

S260 Type XX RTKs: STYK1

S260 Receptor serine/threonine kinase (RSTK) family

S261 Type I receptor serine/threonine kinases

S262 Type II receptor serine/threonine kinases

S262 Type III receptor serine/threonine kinases

S262 RSTK functional heteromers

S264 Receptor tyrosine phosphatase (RTP) family

S266 Tumour necrosis factor (TNF) receptor family

**S272 Enzymes**

S275 Kinases (EC 2.7.x.x)

S276 Rho kinase

S276 Protein kinase C (PKC)

S277 Alpha subfamily

S277 Delta subfamily

S278 Eta subfamily

S278 FRAP subfamily

S279 Cyclin‐dependent kinase (CDK) family

S279 CDK4 subfamily

S279 GSK subfamily

S280 Polo‐like kinase (PLK) family

S280 STE7 family

S281 Abl family

S281 Ack family

S281 Janus kinase (JakA) family

S282 Src family

S283 Tec family

S283 RAF family

S284 Peptidases and proteinases

S284 A1: Pepsin

S284 A22: Presenilin

S285 C14: Caspase

S285 M1: Aminopeptidase N

S285 M2: Angiotensin‐converting (ACE and ACE2)

S286 M10: Matrix metallopeptidase

S286 M12: Astacin/Adamalysin

S287 M28: Aminopeptidase Y

S287 M19: Membrane dipeptidase

S288 S1: Chymotrypsin

S288 T1: Proteasome

S289 S8: Subtilisin

S289 S9: Prolyl oligopeptidase

S290 Acetylcholine turnover

S291 Adenosine turnover

S292 Amino acid hydroxylases

S293 L‐Arginine turnover

S294 2.1.1.‐ Protein arginine N‐methyltransferases

S294 Arginase

S294 Arginine:glycine amidinotransferase

S295 Dimethylarginine dimethylaminohydrolases

S295 Nitric oxide synthases

S296 Carboxylases and decarboxylases

S297 Carboxylases

S298 Decarboxylases

S300 Catecholamine turnover

S302 Ceramide turnover

S303 Serine palmitoyltransferase

S303 Ceramide synthase

S304 Sphingolipid Δ^4^‐desaturase

S304 Sphingomyelin synthase

S305 Sphingomyelin phosphodiesterase

S305 Neutral sphingomyelinase coupling factors

S306 Ceramide glucosyltransferase

S306 Acid ceramidase

S307 Neutral ceramidases

S307 Alkaline ceramidases

S308 Ceramide kinase

S309 Chromatin modifying enzymes

S309 2.1.1.‐ Protein arginine N‐methyltransferases

S310 3.5.1.‐ Histone deacetylases (HDACs)

S310 Cyclic nucleotide turnover/signalling

S310 Adenylyl cyclases (ACs)

S312 Exchange protein activated by cyclic AMP (EPACs)

S312 Nitric oxide (NO)‐sensitive (soluble) guanylyl cyclase

S313 Phosphodiesterases, 3',5'‐cyclic nucleotide (PDEs)

S317 Cytochrome P450

S317 CYP1 family

S318 CYP2 family

S318 CYP3 family

S319 CYP4 family

S320 CYP5, CYP7 and CYP8 families

S320 CYP11, CYP17, CYP19, CYP20 and CYP21 families

S321 CYP24, CYP26 and CYP27 families

S322 CYP39, CYP46 and CYP51 families

S323 Endocannabinoid turnover

S323 *N*‐Acylethanolamine turnover

S324 2‐Acylglycerol ester turnover

S325 Eicosanoid turnover

S325 Cyclooxygenase

S326 Prostaglandin synthases

S327 Lipoxygenases

S328 Leukotriene and lipoxin metabolism

S329 GABA turnover

S331 Glycerophospholipid turnover

S331 Phosphoinositide‐specific phospholipase C

S332 Phospholipase A2

S334 Phosphatidylcholine‐specific phospholipase D

S335 Lipid phosphate phosphatases

S335 Phosphatidylinositol kinases

S336 1‐phosphatidylinositol 4‐kinase family

S336 Phosphatidylinositol‐4‐phosphate 3‐kinase family

S337 Phosphatidylinositol 3‐kinase family

S337 Phosphatidylinositol‐4,5‐bisphosphate 3‐kinase family

S338 1‐phosphatidylinositol‐3‐phosphate 5‐kinase family

S338 Type I PIP kinases (1‐phosphatidylinositol‐4‐phosphate 5‐kinase family)

S339 Type II PIP kinases (1‐phosphatidylinositol‐5‐phosphate 4‐kinase family)

S339 Haem oxygenase

S340 Hydrogen sulphide synthesis

S341 Hydrolases

S342 Inositol phosphate turnover

S342 Inositol 1,4,5‐trisphosphate 3‐kinases

S343 Inositol polyphosphate phosphatases

S343 Inositol monophosphatase

S344 Lanosterol biosynthesis pathway

S346 Nucleoside synthesis and metabolism

S347 Sphingosine 1‐phosphate turnover

S348 Sphingosine kinase

S348 Sphingosine 1‐phosphate phosphatase

S349 Sphingosine 1‐phosphate lyase

S349 Thyroid hormone turnover

S350 1.14.11.29 2‐oxoglutarate oxygenases

S351 1.14.13.9 kynurenine 3‐monooxygenase

S352 2.4.2.30 poly(ADP‐ribose)polymerases

S352 2.5.1.58 Protein farnesyltransferase

S353 3.5.1.‐ Histone deacetylases (HDACs)

S354 3.5.3.15 Peptidyl arginine deiminases (PADI)

S354 RAS subfamily

S355 4.2.1.1 Carbonate dehydratases

S355 5.99.1.2 DNA Topoisomerases

**S360 Transporters**

S362 ATP‐binding cassette transporter family

S362 ABCA subfamily

S363 ABCB subfamily

S365 ABCC subfamily

S366 ABCD subfamily of peroxisomal ABC transporters

S367 ABCG subfamily

S368 F‐type and V‐type ATPases

S368 F‐type ATPase

S368 V‐type ATPase

S369 P‐type ATPases

S369 Na^+^/K^+^‐ATPases

S369 Ca^2+^‐ATPases

S370 H^+^/K^+^‐ATPases

S370 Cu^+^‐ATPases

S370 Phospholipid‐transporting ATPases

S371 Major facilitator superfamily (MFS) of transporters

S371 SLC superfamily of solute carriers

S372 SLC1 family of amino acid transporters

S372 Glutamate transporter subfamily

S374 Alanine/serine/cysteine transporter subfamily

S375 SLC2 family of hexose and sugar alcohol

S375 Class I transporters

S376 Class II transporters

S377 Proton‐coupled inositol transporter

S377 SLC3 and SLC7 families of heteromeric amino acid transporters (HATs)

S377 SLC3 family

S378 SLC7 family

S379 SLC4 family of bicarbonate transporters

S380 Anion exchangers

S380 Sodium‐dependent HCO ${}_{3}^{-}$ transporters

S381 SLC5 family of sodium‐dependent glucose transporters

S381 Hexose transporter family

S382 Choline transporter

S383 Sodium iodide symporter, sodium‐dependent multivitamin transporter and sodium‐coupled monocarboxylate trans‐ porters

S384 Sodium *myo*‐inositol cotransporter transporters

S385 SLC6 neurotransmitter transporter family

S385 Monoamine transporter subfamily

S386 GABA transporter subfamily

S387 Glycine transporter subfamily

S389 Neutral amino acid transporter subfamily

S390 SLC8 family of sodium/calcium exchangers

S390 SLC9 family of sodium/hydrogen exchangers

S391 SLC10 family of sodium‐bile acid co‐transporters

S392 SLC11 family of proton‐coupled metal ion transporters

S393 SLC12 family of cation‐coupled chloride transporters

S395 SLC13 family of sodium‐dependent sulphate/carboxylate transporters

S395 SLC14 family of facilitative urea transporters

S396 SLC15 family of peptide transporters

S398 SLC16 family of monocarboxylate transporters

S399 SLC17 phosphate and organic anion transporter family

S399 Type I sodium‐phosphate co‐transporters

S400 Sialic acid transporter

S400 Vesicular glutamate transporters (VGLUTs)

S401 Vesicular nucleotide transporter

S401 SLC18 family of vesicular amine transporters

S403 SLC19 family of vitamin transporters

S403 SLC20 family of sodium‐dependent phosphate transporters

S404 SLC22 family of organic cation and anion transporters

S404 Organic cation transporters (OCT)

S405 Organic zwitterions/cation transporters (OCTN)

S406 Organic anion transporters (OATs)

S407 Urate transporter

S407 SLC23 family of ascorbic acid transporters

S409 SLC24 family of sodium/potassium/calcium exchangers

S409 SLC25 family of mitochondrial transporters

S410 Mitochondrial di‐ and tri‐carboxylic acid transporter subfamily

S411 Mitochondrial amino acid transporter subfamily

S412 Mitochondrial phosphate transporters

S412 Mitochondrial nucleotide transporter subfamily

S413 Mitochondrial uncoupling proteins

S414 Miscellaneous SLC25 mitochondrial transporters

S414 SLC26 family of anion exchangers

S415 Selective sulphate transporters

S415 Chloride/bicarbonate exchangers

S416 Anion channels

S416 Other SLC26 anion exchangers

S417 SLC27 family of fatty acid transporters

S418 SLC28 and SLC29 families of nucleoside transporters

S418 SLC28 family

S419 SLC29 family

S420 SLC30 zinc transporter family

S421 SLC31 family of copper transporters

S422 SLC32 vesicular inhibitory amino acid transporter

S422 SLC33 acetylCoA transporter

S423 SLC34 family of sodium phosphate co‐transporters

S424 SLC35 family of nucleotide sugar transporters

S425 SLC36 family of proton‐coupled amino acid transporters

S426 SLC37 family of phosphosugar/phosphate exchangers

S427 SLC38 family of sodium‐dependent neutral amino acid transporters

S427 System A‐like transporters

S428 System N‐like transporters

S428 Orphan SLC38 transporters

S429 SLC39 family of metal ion transporters

S430 SLC40 iron transporter

S430 SLC41 family of divalent cation transporters

S431 SLC42 family of Rhesus glycoprotein ammonium transporters

S432 SLC43 family of large neutral amino acid transporters

S433 SLC44 choline transporter‐like family

S433 SLC45 family of putative sugar transporters

S434 SLC46 family of folate transporters

S435 SLC47 family of multidrug and toxin extrusion transporters

S436 SLC48 heme transporter

S436 SLC49 family of FLVCR‐related heme transporters

S437 SLC50 sugar transporter

S438 SLC51 family of steroid‐derived molecule transporters

S438 SLC52 family of riboflavin transporters

S439 SLCO family of organic anion transporting polypeptides

S442 Patched family

## Introduction

In order to allow clarity and consistency in pharmacology, there is a need for a comprehensive organisation and presentation of the targets of drugs. This is the philosophy of the IUPHAR/BPS Guide to PHARMACOLOGY presented on the online free access database (http://www.guidetopharmacology.org/). This database is supported by the British Pharmacological Society (BPS), the International Union of Basic and Clinical Pharmacology (IUPHAR), the University of Edinburgh and previously the Wellcome Trust. Data included in the Guide to PHARMACOLOGY are derived in large part from interactions with the subcommittees of the Nomenclature Committee of the International Union of Basic and Clinical Pharmacology (NC‐IUPHAR). A major influence on the development of the database was Tony Harmar (1951‐2014), who worked with a passion to establish the curators as a team of highly informed and informative individuals, with a focus on high‐quality data input, ensuring a suitably validated dataset. The Editors of the Concise Guide have compiled the individual records, in concert with the team of Curators, drawing on the expert knowledge of these latter subcommittees. The tables allow an indication of the status of the nomenclature for the group of targets listed, usually previously published in Pharmacological Reviews. In the absence of an established subcommittee, advice from several prominent, independent experts has generally been obtained to produce an authoritative consensus on nomenclature, which attempts to fit in within the general guidelines from NC‐IUPHAR. This current edition, the Concise Guide to PHARMACOLOGY 2017/18, is the latest snapshot of the database in print form, following on from the Concise Guide to PHARMACOLOGY 2015/16. It contains data drawn from the online database as a rapid overview of the major pharmacological targets. Thus, there are many fewer targets presented in the Concise Guide compared to the online database. The priority for inclusion in the Concise Guide is the presence of quantitative pharmacological data. This means that often orphan family members are not presented in the Concise Guide, although structural information is available on the online database. The organisation of the data is tabular (where appropriate) with a standardised format, where possible on a single page, intended to aid understanding of, and comparison within, a particular target group. The Concise Guide is intended as an initial resource, with links to additional reviews and resources for greater depth and information. Pharmacological and structural data focus primarily on human gene products, wherever possible, with links to HGNC gene nomenclature and UniProt IDs. In a few cases, where data from human proteins are limited, data from other species are indicated. Pharmacological tools listed are prioritised on the basis of selectivity and availability. That is, agents (agonists, antagonists, inhibitors, activators, etc.) are included where they are both available (by donation or from commercial sources, now or in the near future) AND the most selective. The Concise Guide is divided into nine sections, which comprise pharmacological targets of similar structure/function. These are G protein‐coupled receptors, ligand‐gated ion channels, voltage‐gated ion channels, other ion channels, catalytic receptors, nuclear hormone receptors, enzymes, transporters and other protein targets. We hope that the Concise Guide will provide for researchers, teachers and students a state‐of‐the art source of accurate, curated information on the background to their work that they will use in the Introductions to their Research Papers or Reviews, or in supporting their teaching and studies. We recommend that any citations to information in the Concise Guide are presented in the following format:

Alexander SPH et al. (2017). The Concise Guide to PHARMACOLOGY 2017/18: Overview. Br J Pharmacol 174: S1–S16.

In this overview are listed protein targets of pharmacological interest, which are not G protein‐coupled receptors, ligand‐gated ion channels, voltage‐gated ion channels, ion channels, nuclear hormone receptors, catalytic receptors, transporters or enzymes.

## Acknowledgements

We are extremely grateful to the British Pharmacological Society and the International Union of Basic and Clinical Pharmacology, for financial support of the website and for advice from the NC‐IUPHAR subcommittees. We thank the University of Edinburgh, who host the www.guidetopharmacology.org website. Previously, the International Union of Basic and Clinical Pharmacology and the Wellcome Trust (099156/Z/12/Z]) also supported the initiation and expansion of the database. We are also tremendously grateful to the long list of collaborators from NC‐IUPHAR subcommittees and beyond, who have assisted in the construction of the Concise Guide to PHARMACOLOGY 2017/18 and the online database www.GuideToPHARMACOLOGY.org. Further, we wish to thank Toni Wigglesworth for her assistance in the co‐ordination of correspondence with these collaborators.

## Conflict of interest

The authors state that there are no conflicts of interest to disclose.

## Other Protein Targets

### Family structure

S6 Adiponectin receptors

– B‐cell lymphoma 2 (Bcl‐2) protein family

S7 Blood coagulation components

– Bromodomain‐containing proteins

S7 Non‐enzymatic BRD containing proteins

S8 Carrier proteins

S9 CD molecules

– Chromatin‐interacting transcriptional repressors

S10 Methyllysine reader proteins

– Circadian clock proteins

– Claudins

– EF‐hand domain containing

S11 Fatty acid‐binding proteins

– G‐alpha family G(q) subfamily

– Heat shock proteins

– Immunoglobulins

– Inhibitors of apoptosis (IAP) protein family

– Kelch‐like proteins

– Kinesins

– Leucine‐rich repeat proteins

– Lymphocyte antigens

– Mitochondrial‐associated proteins

– Myosin binding proteins

– Non‐catalytic pattern recognition receptors

– Absent in melanoma (AIM)‐like receptors (ALRs)

– C‐type lectin‐like receptors (CLRs)

– Other pattern recognition receptors

S12 Notch receptors

– Pentaxins

– Serum pentaxins

S13 Regulators of G protein Signaling (RGS) proteins

S14 R4 family

– Repulsive guidance molecules

– Reticulons and associated proteins

– Ribosomal factors

S14 Sigma receptors

S15 Tubulins

– Tumour‐associated proteins

– WD repeat‐containing proteins

## Adiponectin receptors

### Overview

Adiponectin receptors (provisional nomenclature, ENSFM00500000270960) respond to the 30 kDa complement‐related protein hormone adiponectin (also known as ADIPOQ: adipocyte, C1q and collagen domain‐containing protein; ACRP30, adipose most abundant gene transcript 1; apM‐1; gelatin‐binding protein: Q15848) originally cloned from adipocytes [49]. Although sequence data suggest 7TM domains, immunological evidence indicates that, contrary to typical 7TM topology, the carboxyl terminus is extracellular, while the amino terminus is intracellular [90]. Signalling through these receptors appears to avoid G proteins; modelling based on the crystal structures of the adiponectin receptors suggested ceramidase acivity, which would make these the first in a new family of catalytic receptors [93].

**Table:** 

Nomenclature	Adipo1 receptor	Adipo2 receptor
HGNC, UniProt	ADIPOR1, Q96A54	ADIPOR2, Q86V24
Rank order of potency	globular adiponectin (ADIPOQ, Q15848) >adiponectin (ADIPOQ, Q15848)	globular adiponectin (ADIPOQ, Q15848) = adiponectin (ADIPOQ, Q15848)

### Comments

T‐Cadherin (CDH13, P55290) has also been suggested to be a receptor for (hexameric) adiponectin [33].

### Further reading on Adiponectin receptors

Fisman EZ et al. (2014) Adiponectin: a manifold therapeutic target for metabolic syndrome, diabetes, and coronary disease? Cardiovasc Diabetol 13: 103 [PMID:24957699]

Matsuda M et al. (2014) Roles of adiponectin and oxidative stress in obesity‐associated metabolic and cardiovascular diseases. Rev Endocr Metab Disord 15: 1‐10 [PMID:24026768]

Ruan H et al. (2016) Adiponectin signaling and function in insulin target tissues. J Mol Cell Biol 8: 101‐9 [PMID:26993044]

Wang Y et al. (2017) Cardiovascular Adiponectin Resistance: The Critical Role of Adiponectin Receptor Modification. Trends Endocrinol Metab 28: 519‐530 [PMID:28473178]

Zhao L et al. (2014) Adiponectin and insulin cross talk: the microvascular connection. Trends Cardiovasc Med 24: 319‐24 [PMID:25220977]

## Blood coagulation components

### Overview

Coagulation as a process is interpreted as a mechanism for reducing excessive blood loss through the generation of a gel‐like clot local to the site of injury. The process involves the activation, adhesion (see Integrins), degranulation and aggregation of platelets, as well as proteins circulating in the plasma. The coagulation cascade involves multiple proteins being converted to more active forms from less active precursors, typically through proteolysis (see Proteases). Listed here are the components of the coagulation cascade targetted by agents in current clinical usage.

**Table:** 

Nomenclature	coagulation factor V	coagulation factor VIII	serpin family C member 1
HGNC, UniProt	F5, P12259	F8, P00451	SERPINC1, P01008
Selective activators	–	–	heparin (p*K*_d_ 7.8) [26], fondaparinux (p*K*_d_ 7.5) [62], dalteparin [32], danaparoid [16, 56], enoxaparin [19], tinzaparin [20]
Selective inhibitors	drotrecogin alfa [36, 37]	drotrecogin alfa [36, 37]	–

### Further reading on Blood coagulation components

Astermark J. (2015) FVIII inhibitors: pathogenesis and avoidance. Blood 125: 2045‐51 [PMID:25712994]

Girolami A et al. (2017) New clotting disorders that cast new light on blood coagulation and may play a role in clinical practice. J Thromb Thrombolysis 44: 71‐75 [PMID:28251495]

Rana K et al. (2016) Blood flow and mass transfer regulation of coagulation. Blood Rev 30: 357‐68 [PMID:27133256]

## Non‐enzymatic BRD containing proteins

### Overview

Bromodomains bind proteins with acetylated lysine residues, such as histones, to regulate gene transcription. Listed herein are examples of bromodomain‐containing proteins for which sufficient pharmacology exists.

**Table:** 

Nomenclature	bromodomain adjacent to zinc finger domain 2A	bromodomain adjacent to zinc finger domain 2B	CREB binding protein	polybromo 1	SWI/SNF related, matrix associated, actin dependent regulator of chromatin, subfamily a, member 4
HGNC, UniProt	BAZ2A, Q9UIF9	BAZ2B, Q9UIF8	CREBBP, Q92793	PBRM1, Q86U86	SMARCA4, P51532
Selective inhibitors	GSK2801 (p*K*_d_ 6.6) [73]	GSK2801 (p*K*_d_ 6.9) [73]	I‐CBP112 (p*K*_d_ 6.8) [72]	PFI‐3 (p*K*_d_ 7.3) [79]	PFI‐3 (p*K*_d_ 7.1) [79]

### Further reading on Non‐enzymatic BRD containing proteins

Brand M et al. (2015) Small molecule inhibitors of bromodomain‐acetyl‐lysine interactions. ACS Chem. Biol. 10: 22‐39 [PMID:25549280]

Fujisawa T et al. (2017) Functions of bromodomain‐containing proteins and their roles in homeostasis and cancer Nat Rev Mol Cell Biol 18: 246‐262 [PMID:28053347]

Nicholas DA et al. (2017) BET bromodomain proteins and epigenetic regulation of inflammation: implications for type 2 diabetes and breast cancer. Cell Mol Life Sci 74: 231‐243 [PMID:27491296]

Theodoulou NH et al. (2016) Clinical progress and pharmacology of small molecule bromodomain inhibitors. Curr Opin Chem Biol 33: 58‐66 [PMID:27295577]

Theodoulou NH et al. (2016) Progress in the Development of non‐BET Bromodomain Chemical Probes. ChemMedChem 11: 477‐87 [PMID:26749027]

## Carrier proteins

### Overview

Transthyretin (TTR) is a homo‐tetrameric protein which transports thyroxine in the plasma and cerebrospinal fluid and retinol (vitamin A) in the plasma. Many disease causing mutations in the protein have been reported, many of which cause complex dissociation and protein mis‐assembly and deposition of toxic aggregates amyloid fibril formation [63]. These amyloidogenic mutants are linked to the development of pathological amyloidoses, including familial amyloid polyneuropathy (FAP) [4, 14], familial amyloid cardiomyopathy (FAC) [34], amyloidotic vitreous opacities, carpal tunnel syndrome [54] and others. In old age, non‐mutated TTR can also form pathological amyloid fibrils [88]. Pharmacological intervention to reduce or prevent TTR dissociation is being pursued as a theapeutic strategy. To date one small molecule kinetic stabilising molecule (tafamidis) has been approved for FAP, and is being evaluated in clinical trials for other TTR amyloidoses.

**Table:** 

Nomenclature	transthyretin
HGNC, UniProt	TTR, P02766
Common abreviation	TTR

### Further reading on Carrier proteins

Alshehri B et al. (2015) The diversity of mechanisms influenced by transthyretin in neurobiology: development, disease and endocrine disruption. J Neuroendocrinol 27: 303‐23 [PMID:25737004]

Delliere S et al. (2017) Is transthyretin a good marker of nutritional status? Clin Nutr 36: 364‐370 [PMID:27381508]

Galant NJ et al. (2017) Transthyretin amyloidosis: an under‐recognized neuropathy and cardiomyopathy. Clin Sci (Lond) 131: 395‐409 [PMID:28213611]

## CD molecules

### Overview

Cluster of differentiation refers to an attempt to catalogue systematically a series of over 300 cell‐surface proteins associated with immunotyping. Many members of the group have identified functions as enzymes (for example, see CD73 ecto‐5'‐nucleotidase) or receptors (for example, see CD41 integrin, alpha 2b subunit). Many CDs are targetted for therapeutic gain using antibodies for the treatment of proliferative disorders. A full listing of all the Clusters of Differentiation is not possible in the Guide to PHARMACOLOGY; listed herein are selected members of the family targetted for therapeutic gain.

**Table:** 

Nomenclature	CD2	CD3e	CD20 (membrane‐spanning 4‐domains, subfamily A, member 1)	CD33	CD52
HGNC, UniProt	CD2, P06729	CD3E, P07766	MS4A1, P11836	CD33, P20138	CD52, P31358
Common abreviation	–	–	–	SIGLEC‐3	–
Selective inhibitors	alefacept (Inhibition) [17, 53]	–	–	–	–
Antibodies	–	catumaxomab (Binding) [43], muromonab‐CD3 (Binding) [25], otelixizumab (Binding) [9]	ofatumumab (Binding) (p*K*_d_ 9.9) [47], rituximab (Binding) (p*K*_d_ 8.5) [75], ibritumomab tiuxetan (Binding), obinutuzumab (Binding) [3, 66], tositumomab (Binding)	lintuzumab (Binding) (p*K*_d_∼10) [10], gemtuzumab ozogamicin (Binding) [7]	alemtuzumab (Binding) [24, 79]

**Table:** 

Nomenclature	CD80	CD86	cytotoxic T‐lymphocyte‐associated protein 4 (CD152)	programmed cell death 1 (CD279)	CD300a
HGNC, UniProt	CD80, P33681	CD86, P42081	CTLA4, P16410	PDCD1, Q15116	CD300A, Q9UGN4
Common abreviation	–	–	CTLA‐4	PD‐1	–
Antibodies	–	–	ipilimumab (p*K*_d_>9) [28], tremelimumab (p*K*_d_ 8.9) [30]	pembrolizumab (p*K*_d_∼10) [11], nivolumab (p*K*_d_ 9.1) [28, 38, 40]	–

### Comment

The endogenous ligands for human PD‐1 are programmed cell death 1 ligand 1 (PD‐L1 aka CD274(CD274, Q9NZQ7)) and programmed cell death 1 ligand 2 (PD‐L2; PDCD1LG2). These ligands are cell surface peptides, normally involved in immune system regulation. Expression of PD‐1 by cancer cells induces immune tolerance and evasion of immune system attack. Anti‐PD‐1 monoclonal antibodies are used to induce immune checkpoint blockade as a therapeutic intervention in cancer, effectively re‐establishing immune vigilance. pembrolizumab was the first anti‐PD‐1 antibody to be approved by the US FDA.

### Further reading on CD molecules

Gabius HJ et al. (2015) The glycobiology of the CD system: a dictionary for translating marker designations into glycan/lectin structure and function. Trends Biochem Sci 40: 360‐76 [PMID:25981696]

## Methyllysine reader proteins

### Overview

Methyllysine reader proteins bind to methylated proteins, such as histones, allowing regulation of gene expression.

**Table:** 

Nomenclature	l(3)mbt‐like 3 (Drosophila)
HGNC, UniProt	L3MBTL3, Q96JM7
Selective agonists	UNC1215 [35]

### Further reading on Methyllysine reader proteins

Liu K et al. (2015) Epigenetic targets and drug discovery Part 2: Histone demethylation and DNA methylation. Pharmacol. Ther. 151: 121‐40 [PMID:25857453]

Milosevich N et al. (2016) Chemical Inhibitors of Epigenetic Methyllysine Reader Proteins. Biochemistry 55: 1570‐83 [PMID:26650180]

Sadakierska‐Chudy A et al. (2015) A comprehensive view of the epigenetic landscape part I: DNA methylation, passive and active DNA demethylation pathways and histone variants. Neurotox Res 27: 84‐97 [PMID:25362550]

Teske KA et al. (2017) Methyllysine binding domains: Structural insight and small molecule probe development. Eur J Med Chem 136: 14‐35 [PMID:28478342]

Zahnow CA et al. (2016) Inhibitors of DNA Methylation, Histone Deacetylation, and Histone Demethylation: A Perfect Combination for Cancer Therapy. Adv Cancer Res 130: 55‐111 [PMID:27037751]

## Fatty acid‐binding proteins

### Overview

Fatty acid‐binding proteins are low molecular weight (100‐130 aa) chaperones for long chain fatty acids, fatty acyl CoA esters, eicosanoids, retinols, retinoic acids and related metabolites and are usually regarded as being responsible for allowing the otherwise hydrophobic ligands to be mobile in aqueous media. These binding proteins may perform functions extracellularly (e.g. in plasma) or transport these agents; to the nucleus to interact with nuclear receptors (principally PPARs and retinoic acid receptors [70]) or for interaction with metabolic enzymes. Although sequence homology is limited, crystallographic studies suggest conserved 3D structures across the group of binding proteins.

**Table:** 

Nomenclature	fatty acid binding protein 1	fatty acid binding protein 2	fatty acid binding protein 3	fatty acid binding protein 4
HGNC, UniProt	FABP1, P07148	FABP2, P12104	FABP3, P05413	FABP4, P15090
Rank order of potency	stearic acid, oleic acid>palmitic acid, linoleic acid>arachidonic acid, *α*‐linolenic acid [67]	stearic acid>palmitic acid,oleic acid>linoleic acid>arachidonic acid, *α*‐linolenic acid [67]	stearic acid, oleic acid, palmitic acid>linoleic acid, *α*‐linolenic acid, arachidonic acid [67]	oleic acid, palmitic acid, stearic acid, linoleic acid>*α*‐linolenic acid, arachidonic acid [67]
Inhibitors	fenofibrate (p*K*_i_ 7.6) [12] – Rat, fenofibric acid (p*K*_i_ 6.5) [12] – Rat, HTS01037 (p*K*_i_ 5.1) [30] – Mouse	–	–	–
Selective inhibitors	–	–	–	HM50316 (p*K*_i_>9) [46]
Comments	A broader substrate specificity than other FABPs, binding two fatty acids per protein [82].	Crystal structure of the rat FABP2 [69].	Crystal structure of the human FABP3 [91].	–

**Table:** 

Nomenclature	fatty acid binding protein 5	fatty acid binding protein 6	fatty acid binding protein 7	peripheral myelin protein 2	fatty acid binding protein 9	fatty acid binding protein 12
HGNC, UniProt	FABP5, Q01469	FABP6, P51161	FABP7, O15540	PMP2, P02689	FABP9, Q0Z7S8	FABP12, A6NFH5
Comments	Crystal structure of the human FABP5 [31].	Able to transport bile acids [95].	Crystal structure of the human FABP7 [5].	In silico modelling suggests that PMP2/FABP8 can bind both fatty acids and cholesterol [50].	–	–

**Table:** 

Nomenclature	retinol binding protein 1	retinol binding protein 2	retinol binding protein 3	retinol binding protein 4	retinol binding protein 5	retinol binding protein 7
HGNC, UniProt	RBP1, P09455	RBP2, P50120	RBP3, P10745	RBP4, P02753	RBP5, P82980	RBP7, Q96R05
Rank order of potency	–	stearic acid>palmitic acid, oleic acid, linoleic acid, *α*‐linolenic acid, arachidonic acid [68]	–	–	–	–
Inhibitors	–	–	–	A1120 (pIC_50_ 7.8) [86]	–	–

**Table:** 

Nomenclature	retinaldehyde binding protein 1	cellular retinoic acid binding protein 1	cellular retinoic acid binding protein 2
HGNC, UniProt	RLBP1, P12271	CRABP1, P29762	CRABP2, P29373
Rank order of potency	11‐cis‐retinal, 11‐cis‐retinol>9‐cis‐retinal, 13‐cis‐retinal, 13‐cis‐retinol, all‐trans‐retinal, retinol [15]	tretinoin>alitretinoin stearic acid>palmitic acid, oleic acid, linoleic acid, *α*‐linolenic acid, arachidonic acid [68]	–

### Comments

Although not tested at all FABPs, BMS309403 exhibits high affinity for FABP4 (pIC50 ˜8.8) compared to FABP3 or FABP5 (pIC50 <6.6) [21, 81]. HTS01037 is reported to interfere with FABP4 action [30]. Ibuprofen displays some selectivity for FABP4 (pIC_50_ 5.5) relative to FABP3 (pIC_50_ 3.5) and FABP5 (pIC_50_ 3.8) [48]. Fenofibric acid displays some selectivity for FABP5 (pIC_50_ 5.5) relative to FABP3 (pIC_50_ 4.5) and FABP4 (pIC_50_ 4.6) [48]. Multiple pseudogenes for the FABPs have been identified in the human genome.

### Further reading on Fatty acid‐binding proteins

Gajda AM et al. (2015) Enterocyte fatty acid‐binding proteins (FABPs): different functions of liver and intestinal FABPs in the intestine. Prostaglandins Leukot. Essent. Fatty Acids 93: 9‐16 [PMID:25458898]

Glatz JF. (2015) Lipids and lipid binding proteins: a perfect match. Prostaglandins Leukot. Essent. Fatty Acids 93: 45‐9 [PMID:25154384]

Hotamisligil GS et al. (2015) Metabolic functions of FABPs‐mechanisms and therapeutic implications. Nat Rev Endocrinol 11: 592‐605 [PMID:26260145]

Matsumata M et al. (2016) Fatty acid binding proteins and the nervous system: Their impact on mental conditions. Neurosci. Res. 102: 47‐55 [PMID:25205626]

Osumi T et al. (2016) Heart lipid droplets and lipid droplet‐binding proteins: Biochemistry, physiology, and pathology. Exp Cell Res 340: 198‐204 [PMID:26524506]

## Notch receptors

### Overview

The canonocal Notch signalling pathway has four type I transmembrane Notch receptors (Notch1‐4) and five ligands (DLL1, 2 and 3, and Jagged 1‐2). Each member of this highly conserved receptor family plays a unique role in cell‐fate determination during embryogenesis, differentiation, tissue patterning, proliferation and cell death [2]. As the Notch ligands are also membrane bound, cells have to be in close proximity for receptor‐ligand interactions to occur. Cleavage of the intracellular domain (ICD) of activated Notch receptors by *γ*‐secretase is required for downstream signalling and Notch‐induced transcriptional modulation [18, 57, 71, 89]. This is why *γ*‐secretase inhibitors can be used to downregulate Notch signalling and explains their anti‐cancer action. One such small molecule is RO4929097[47], although development of this compound has been terminated following an unsuccessful Phase II single agent clinical trial in metastatic colorectal cancer [78].

Aberrant Notch signalling is implicated in a number of human cancers [41, 59, 74, 85]. Pharmaceutical inhibitors of Notch signalling such as demcizumab and tarextumab are being actively investigated as novel anti‐cancer agents [64].

**Table:** 

Nomenclature	notch 1	notch 2	notch 3	notch 4
HGNC, UniProt	NOTCH1, P46531	NOTCH2, Q04721	NOTCH3, Q9UM47	NOTCH4, Q99466
Comments	Various types of activating and inactivating NOTCH1 mutations have been reported to be associated with human diseases, for example: aortic valve disease [23, 52], Adams‐Oliver syndrome 5 [76], T‐cell acute lymphoblastic leukemia (T‐ALL) [87], chronic lymphocytic leukemia (CLL) [65] and head and neck squamous cell carcinoma [1, 77].	–	–	Notch 4 is a potential therapeutic molecular target for triple‐negative breast cancer [42, 55].

### Further reading on Notch receptors

Borggrefe T et al. (2016) The Notch intracellular domain integrates signals from Wnt, Hedgehog, TGFbeta/BMP and hypoxia pathways. Biochim Biophys Acta 1863: 303‐313 [PMID:26592459]

Cheng YL et al. (2015) Emerging roles of the gamma‐secretase‐notch axis in inflammation. Pharmacol Ther 147: 80‐90 [PMID:25448038]

Palmer WH et al. (2015) Ligand‐Independent Mechanisms of Notch Activity. Trends Cell Biol 25: 697‐707 [PMID:26437585]

Previs RA et al. (2015) Molecular pathways: translational and therapeutic implications of the Notch signaling pathway in cancer. Clin Cancer Res 21: 955‐61 [PMID:25388163]

Takebe N et al. (2015) Targeting Notch, Hedgehog, and Wnt pathways in cancer stem cells: clinical update. Nat Rev Clin Oncol 12: 445‐464 [PMID:25850553]

## Regulators of G protein Signaling (RGS) proteins

### Overview

Regulators of G protein signalling (RGS) proteins increase the deactivation rates of G protein signalling pathways through enhancing the GTPase activity of the G protein alpha subunit. Interactions through protein:protein interactions of many RGS proteins have been identified for targets other than heteromeric G proteins. The 20 RGS proteins are commonly divided into four families (R4, R7, R12 and RZ) based on sequence and domain homology. Described here is RGS4 for which a number of pharmacological inhibitors have been described.

**Table:** 

Nomenclature	regulator of G‐protein signaling 4
HGNC, UniProt	RGS4, P49798
Common abreviation	RGS4
Selective inhibitors	RGS4 inhibitor 11b (pIC_50_ 7.8) [83], CCG‐50014 (pIC_50_ 7.5) [8, 83], RGS4 inhibitor 13 (pIC_50_ 7.3) [83]

### Further reading on RGS proteins

Sethakorn N et al. (2010) Non‐canonical functions of RGS proteins. Cell Signal 22: 1274‐81 [PMID:20363320]

Sjogren B (2017) The evolution of regulators of G protein signalling proteins as drug targets ‐ 20 years in the making: IUPHAR Review 21. Br J Pharmacol 174: 427‐437 [PMID:28098342]

Sjogren B et al. (2010) Thinking outside of the "RGS box": new approaches to therapeutic targeting of regulators of G protein signaling. Mol Pharmacol 78: 550‐7 [PMID:20664002]

Turner EM et al. (2012) Small Molecule Inhibitors of Regulator of G Protein Signalling (RGS) Proteins. ACS Med Chem Lett 3: 146‐150 [PMID:22368763]

## Sigma receptors

### Overview

Although termed ‘receptors’, the evidence for coupling through conventional signalling pathways is lacking. Initially described as a subtype of opioid receptors, there is only a modest pharmacological overlap and no structural convergence with the G protein‐coupled receptors; the crystal structure of the sigma1 receptor [94] suggests a trimeric structure of a single short transmembrane domain traversing the endoplasmic reticulum membrane, with the bulk of the protein facing the cytosol. A wide range of compounds, ranging from psychoactive agents to antihistamines, have been observed to bind to these sites.

**Table:** 

Nomenclature	sigma non‐opioid intracellular receptor 1	*σ*2
HGNC, UniProt	SIGMAR1, Q99720	–
Selective agonists	PRE‐084 [80], (+)‐SKF 10.047	–
Selective antagonists	NE‐100 (pIC_50_ 8.4) [60], BD‐1047 (pIC_50_ 7.4) [51]	–
Labelled ligands	[^3^H]pentazocine (Agonist)	[^3^H]‐di‐o‐tolylguanidine (Agonist)

### Comments

(‐)‐pentazocine also shows activity at opioid receptors. The sigma2 receptor has recently been reported to be TMEM97 Q5BJF2[92] , a 4TM protein partner of NPC1, the Niemann‐Pick C1 protein, a 13TM cholesterol‐binding protein.

### Further reading on Sigma receptors

Chu UB et al. (2016) Biochemical Pharmacology of the Sigma‐1 Receptor. Mol Pharmacol 89: 142‐53 [PMID:26560551]

Gris G et al. (2015) Sigma‐1 receptor and inflammatory pain. Inflamm Res 64: 377‐81 [PMID:25902777]

Rousseaux CG et al. (2015) Sigma receptors [sigmaRs]: biology in normal and diseased states. J Recept Signal Transduct Res 1‐62 [PMID:26056947]

Su TP et al. (2016) The Sigma‐1 Receptor as a Pluripotent Modulator in Living Systems. Trends Pharmacol Sci 37: 262‐78 [PMID:26869505]

van Waarde A et al. (2015) Potential applications for sigma receptor ligands in cancer diagnosis and therapy. Biochim Biophys Acta 1848: 2703‐14 [PMID:25173780]

## Tubulins

### Overview

Tubulins are a family of intracellular proteins most commonly associated with microtubules, part of the cytoskeleton. They are exploited for therapeutic gain in cancer chemotherapy as targets for agents derived from a variety of natural products: taxanes, colchicine and vinca alkaloids. These are thought to act primarily through *β*‐tubulin, thereby interfering with the normal processes of tubulin polymer formation and disassembly.

**Table:** 

Nomenclature	tubulin alpha 1a	tubulin alpha 4a	tubulin beta class I	tubulin beta 3 class III	tubulin beta 4B class IVb	tubulin beta 8 class VIII
HGNC, UniProt	TUBA1A, Q71U36	TUBA4A, P68366	TUBB, P07437	TUBB3, Q13509	TUBB4B, P68371	TUBB8, Q3ZCM7
Inhibitors	–	–	vinblastine(pIC_50_ 9), vincristine, eribulin(pIC_50_ 8.2) [58], paclitaxel(pEC_50_ 8.1) [61], colchicine(pIC_50_ 8) [13], cabazitaxel, docetaxel, ixabepilone	combretastatin A4(pIC_50_ 8.2) [22]	–	–

### Further reading on Tubulins

Gadadhar S et al. (2017) The tubulin code at a glance. J Cell Sci 130: 1347‐1353

Penna LS et al. (2017) Anti‐mitotic agents: Are they emerging molecules for cancer treatment? Pharmacol Ther 173: 67‐82 [PMID:28174095]
